# ﻿A new North American species of *Etainia* (Lepidoptera, Nepticulidae), feeding on *Arbutus* and *Arctostaphylos* species (Ericaceae)

**DOI:** 10.3897/zookeys.1193.116982

**Published:** 2024-03-07

**Authors:** Erik J. van Nieukerken, Donald R. Davis, Steven V. Swain, Marc E. Epstein

**Affiliations:** 1 Naturalis Biodiversity Center, PO Box 9557, NL-2300 RA Leiden, Netherlands Naturalis Biodiversity Center Leiden Netherlands; 2 Department of Entomology, National Museum of Natural History, MRC 105, Smithsonian Institution, PO Box 37012, Washington, DC 20013–7012, USA National Museum of Natural History, Smithsonian Institution Washington, DC United States of America; 3 Environmental Horticulture Advisor Marin & Sonoma Counties, 1682 Novato Blvd., Suite 150-B, Novato, CA 94947, USA Environmental Horticulture Advisor Marin & Sonoma Counties Novato, CA United States of America; 4 Plant Pest Diagnostics Center, California Department of Food & Agriculture, 3294 Meadowview Rd., Sacramento, CA 95832, USA Plant Pest Diagnostics Center, California Department of Food & Agriculture Sacramento United States of America

**Keywords:** Arizona, California, Canada, insect damage, leafmines, stem mines, taxonomy, United States

## Abstract

*Etainiathoraceleuca* van Nieukerken, Epstein & Davis, **sp. nov.** is the second native American species of *Etainia* Beirne, 1945, and the second known *Etainia* species feeding on Ericaceae. The species is known from light-collected adults in the USA (California, Arizona) and Canada (Ontario). These were linked via DNA barcodes to larvae that make short leafmines on *Arbutus* and *Arctostaphylos* species, then continue feeding in stems and branches, causing damage in nurseries and planted trees in Sonoma and Marin Counties, California. The holotype was accidentally reared from *Arbutusarizonica*, without observing the damage. Life history and damage are described in detail. Damage in *Arctostaphylosuva-ursi* found in Washington State probably belongs to *E.thoraceleuca*, which is a sister species to the European *E.albibimaculella* (Larsen, 1927).

## ﻿Introduction

Trees of the genus *Arbutus* are popular as planted trees for landscaping and gardening in western North America; this applies both to the native Pacific madrone (*A.menziesii* Pursh) as well as to the European Strawberry tree (*A.unedo* L.) and the cultivar *Arbutus* ‘Marina’, which is of obscure hybrid origin, probably from European stock (San Marcos Growers 2023). Especially the latter is frequently planted as street trees, for example, in San Francisco (Martin et al. 2016). Several Lepidoptera are known as leafminers of *Arbutus*, especially the heliozelid *Coptodiscaarbutiella* Busck, 1904 and the gracillariid *Marmaraarbutiella* Busck, 1903, which are common on Pacific madrone (Hunt et al. 1992; Eiseman 2022). In 2014 nurseries in Marin County, California reported unknown damage on *Arbutus*, causing leaf spots and dieback of shoots (Zwart 2017). This damage was first investigated by the late Steven Seybold (University of California - Davis), Drew Zwart, and SVS, who eventually sent larval samples to EvN. DNA barcoding matched these to an undescribed species of *Etainia* (Nepticulidae) that had been known for some time from adults collected at light, and one specimen that was accidentally reared by D.L. Wagner from material collected on *Arbutusarizonica* by R.S. Wielgus in 1987.

The Nepticulidae are a medium–large family of very small moths, of which the majority make leafmines as larvae. Globally around 1000 species are known, and 97 named species occur in North America, but many are still unnamed (van Nieukerken et al. 2016; van Nieukerken and Eiseman 2023).

The genus *Etainia* Beirne, 1945 is one of the smaller nepticulid genera with only 17 named species from Asia, Europe, Africa, and North America (van Nieukerken et al. 2016; Yagi and Hirowatari 2017) and around five undescribed species known in collections. The genus has also been known under the preoccupied name Obrussa Braun, 1915 or as a subgenus of Ectoedemia Busck, 1907. It is one of the few genera of which none of the species make the typical nepticulid leafmines but rather feed in shoots, buds, or fruits. Known hosts belong to Sapindaceae: *Acer* and Ericaceae, but the hosts for most species are still unknown. The hitherto only known native North American species, *E.ochrefasciella* (Chambers, 1873), feeds in petioles and buds of sugar maple (*Acersaccharum* Marshall) and its subspecies (Kulman 1967). The European *E.sericopeza* (Zeller, 1839), now a widespread alien in North America, feeds in summer in the keys (samaras) of Norway maple, *A.platanoides* L., and in winter also in a similar fashion as *E.ochrefasciella* (Jäckh 1951; Emmet and Johnson 1977). In North America it has also been reported to mine petioles of the host in summer (Felt 1930).

The European *E.albibimaculella* (Larsen, 1927) feeds on bearberry (or kinnikinnick), *Arctostaphylosuva-ursi* (L.) Spreng., and makes mines that start in the leaf but then immediately enter the shoots (Adamczewski 1947). The specimen of the new species, taken in Almonte, Ontario, in an area where bearberry is common, was first misidentified as *E.albibimaculella*, which as a result was wrongly recorded as Holarctic and Canadian (van Nieukerken et al. 2016; van Nieukerken 2018).

Adults in the genus *Etainia* can be best recognized by the long apodemes on the dorsal surface of the valvae in the male genitalia (e.g. Fig. 7). More apomorphies and general descriptions are provided by van Nieukerken (1986, 2020), van Nieukerken and Johansson (1990), Puplesis and Diškus (1996), van Nieukerken and Laštůvka (2002), Yagi and Hirowatari (2017), and Shin et al. (2022).

We here describe the new species and compare it with congeners and other similar North American species. The other two North American species were previously treated by Wilkinson and Scoble (1979).

## ﻿Material and methods

### ﻿Material

We list here material without coordinates, more detailed specimen data are provided in GBIF dataset https://doi.org/10.15468/espa8k.

#### ﻿Abbreviations for depositories, etc.(often combined with catalog numbers)

**BIN** Barcode Index Number (Ratnasingham and Hebert 2013);

**BIOUG**Biodiversity Institute of Ontario, University of Guelph, ON, Canada;

**BOLD** Barcode of Life Data Systems (http://www.barcodinglife.com/);

**CASENT**California Academy of Sciences, Entomology, San Francisco, CA, USA;

**CSCA**California State Collection of Arthropods, Sacramento, CA, USA;

**CNC**Canadian National Collection of Insects, Arachnids and Nematodes, Ottawa, ON, Canada;

**EMEC**Essig Museum of Entomology, Berkeley, CA, USA;

**MZH**Luomus, Finnish Museum of Natural History, Helsinki, Finland;

**RMNH**Naturalis Biodiversity Center, Zoological collections, Leiden, The Netherlands;

**USNM**National Museum of Natural History, Smithsonian Institution, Washington DC, USA.

### ﻿Methods

Adults were usually collected at light by various collectors. The holotype was reared accidentally. SVS collected adults by setting five-gallon *Arbutus* ‘Marina’ (bush) trap plants out nearby infected trees from 2900 Wild Turkey Run, in the Bennett Valley area south of Santa Rosa, California. Once trap plants showed symptoms of infestation, they were transported back to a holding area, where the plants had plastic funnels constructed around their bases (0.013 mm clear polycarbonate sheeting cut and glued into a 68 cm tall funnel, with a top opening of 46 cm and a bottom opening of 25 cm). Nets measuring 175 cm × 71 cm were constructed using a Singer model 4423 sewing machine, of white organdy (JoAnn Fabrics and Crafts, Rohnert Park, CA), white polyester thread, and cinched with black 4 mm diameter parachute cord. These nets were placed over the entire plant and funnel assembly in April of 2020 and checked twice weekly.

Larvae were collected by cutting off ends of symptomatic branches of various hostplants and dissecting them using a scalpel under a Leica MZ75 stereomicroscope. Larvae were teased from their tunnels with a pin, and frozen before shipment to CDFA labs, or alternatively kept in ethanol 80%.

Pupae were recovered from the duff within the adult enclosures (above) and later from beneath heavily infested plants in the landscape. Cocoons were found about 5 cm beneath the surface of the duff, usually sandwiched between fragments of two dead leaves to which they were lightly attached.

We further added data obtained from observation platforms iNaturalist, BugGuide, and Barcode of Life Data Systems.

#### ﻿Morphology

Genitalia were prepared according to standard procedures—those by DRD using Canada balsam as embedding medium, those by EvN usually including DNA extraction—and using Euparal as embedding medium; see earlier papers (van Nieukerken 1985; van Nieukerken et al. 2010). Larval slides were prepared in the same way.

#### ﻿Measurements

Measurements of genitalia were obtained from digital images, using calibrated scaling in the Zeiss AxioVision software; we used a 20× objective for male genitalia and 10× or 20× for female genitalia. Capsule length was measured from vinculum to middle of pseuduncus; valva length from tip of posterior process to ventral edge, excluding the sublateral process; phallus length was measured along the sclerotized tube, from tip, excluding carinae. Total corpus bursa length was measured from where the ductus bursae widens into the corpus bursae to anterior edge of bursa. Genitalia measurements are rounded off to the nearest 5 μm. Forewing length was measured from tip of fringe to attachment on thorax, with a Zeiss SV11 stereomicroscope at a magnification of 20×. Antennal segment counts include scape and pedicel; they were counted on photographs or directly under the same stereo microscope. Larval measurements of potential 2^nd^, 3^rd^, and 4^th^ instars, mounted on slides, were from San Rafael, California (Figs 22, 23, 26) (S. Seybold, see below) and those measured from ethanol by MEE were 1^st^ to 4^th^ instars (*n* = 21) and from San Rafael and Sonoma, California (Figs 18–21, 24, 25, 27, 28; see below).

#### ﻿Photographs

Photographs of moths were made with an AxioCam MRc 5 digital camera attached to a motorized Zeiss SteREO Discovery V12, using the Module Extended Focus and Zeiss AxioVision software to prepare a picture in full focus from a Z-stack of ca 10–40 individual photos. Genitalia were photographed with an MRc 5 camera on a manually operated Zeiss Axioskop H, without using extended focus. Photographs were edited with Adobe Photoshop (various versions), avoiding changes to the real object, but backgrounds were cleaned of excess debris and artifacts by using the healing brush and clone tools; tone and contrast are adjusted, and some sharpening was used. Larvae in fluid, as well as dry cocoons, pupal skin, and a parasitoid, were photographed with a Leica MZ 16 using LAS IV Z-stack, as above, and scanning electron microscopy of the larvae was done using a Vega 3 Tescan with normal vacuum; the samples were sputter-coated with gold palladium.

#### ﻿DNA barcoding

Our methodology has been described in other papers (van Nieukerken et al. 2012; Doorenweerd et al. 2015, 2016). We present a neighbor-joining tree, with KP2 distances, of the selected taxa, made with tools provided by BOLD Systems (Ratnasingham and Hebert 2007). The DNA barcode data as used here are given in detail in the public BOLD dataset DS-ETAARB (*Etainia*Arbutus) (https://doi.org/10.5883/DS-ETAARB), including GenBank accession numbers.

#### ﻿Hostplants

Hostplant names follow Catalogue of Life (Hassler 2023) and the Flora of North America (Parker et al. 2009; Sørensen 2009). The larvae were recovered from various manzanita species (*Arctostaphylos*). Given the large number of species in the genus, particularly in California, we did not attempt to identify the species of hostplant, but some of the cited observations do include species identifications. See Parker et al. (2009) for more information. Where species names are provided, we cannot guarantee that all species identifications are fully correct.

## ﻿Results

### ﻿ Etainia thoraceleuca

Taxon classificationAnimaliaLepidopteraNepticulidae

van Nieukerken, Epstein & Davis sp. nov.

29209AF2-0DD3-5F22-B6A6-E4C2449F6EF7

https://zoobank.org/C483BF94-3D5F-4AFE-9336-40FFEDAD37C3

Etainia albibimaculella ; van Nieukerken et al. 2016: 145; van Nieukerken 2018: 32 [North American, Canadian records, misidentification].

#### Type material.

***Holotype*.** United States • ♂; Arizona, Cochise Co., Huachuca Mts., Miller Canyon; 31.4248, −110.26; 5,200’ [1585 m]; 19.iii.1987; larva collected with *Arbutusarizonica*; R. S. Wielgus leg.; emerged 24.v.1987 [reared by D.L. Wagner], DLW Lot: 87C4.5; Genitalia slide EvN4950; USNM01850751.

***Paratypes*** (13♂, 6♀). United States – **Arizona** • 1♂; Cochise Co., Ash Canyon, Huachuca Mts.; 5100’ [1550 m]; 6.x.1979; P.M. Jump leg.; Genitalia slide USNM16408; USNM01850740 • 1♂; Yavapai Co., 20 km W. Prescott, Yavapai CG [Campground]; 9.vi.1997; oak-juniper-pine; H.W. van der Wolf leg.; Genitalia slide EvN5492; RMNH.INS.25492. – **California**: 1♂ (abdomen missing); Contra Costa Co., Walnut Creek; 14.x.1961; J. Powell leg.; USNM01850749 • 1♂; Del Norte Co., Grassy Flat Campground, 3 mi. [4.8 km] W Patrick’s Creek; 2.x.1968; P.A. Opler leg.; at light; Genitalia slide USNM20961; USNM01850741 • 1♂; Lake Co., Pogie Point, Lake Pillsbury; 28–29.viii.1973; J. Powell leg.; at light; Genitalia slide DRD3151; USNM01850742 • 1♂; Los Angeles Co., San Gabriel Mts., San Gabriel Canyon, Red Box Canyon Road; 31.viii.1974; black lite; D.C. Frack leg.; USNM01850743 • 1♂; Madera Co., O’Neals; 26.iv.2015; V. + M. Albu leg.; Genitalia slide EvN4998; RMNH.INS.24998 • 1♂ (abdomen missing); Medocino Co., South of Piercy; 9.viii.1971, 10.viii.1971; R.H. Leuschner leg.; USNM01850750 • 1 ♂; same collecting data; EMEC752122 • 1♂; Riverside Co, San Jacinto Mts, Keen Camp; 4,500’ [1370 m]; 31.viii.1974; D.C. Frack leg.; black lite; Genitalia slide USNM20877; USNM01850744 • 1♂; San Bernardino Co., Wild Horse Canyon, Rt. 2, 0.7 mi. [1.13 km] W Jct. Rt. 138; 4,800’ [1465 m]; 21.vi.1974; D. Frack leg.; black lite; Genitalia slide DRD3403; USNM01850745 • 1♀; same collecting data; 23.vii.1975; Genitalia slide DRD3401; USNM01850746 • 1♂ [in ethanol]; San Diego Co., San Dieguito River Valley Conservancy, Site 1 (Volcan Mtn); 24.viii.2013; Joshua Kohn leg.; T, Upland, Malaise trap; Genitalia slide EvN4941; BIOUG08839-B12 • 2♀; Santa Barbara Co., 1 mi. NE San Marcos Pass; 1500’ [460 m]; 7.vii.1965; J. Powell leg.; Genitalia slide USNM17493; EMEC1744182, EMEC1744183 • 1♀; Santa Barbara Co., 2 mi. [3.2 km] W Los Prietos; 7.ix.1969; P. Opler leg.; at light; Genitalia slide DRD3330; USNM01850747 • 1♀; Siskiyou Co., Happy Camp; 8.vii.1958; J. Powell leg.; at light; Genitalia slide DRD3329; USNM01850748 • 1♂; Siskiyou Co., 8 km SW Mount Shasta, Castle Lake; 12.viii.1992; H.W. van der Wolf leg.; Genitalia slide EvN4951; RMNH.INS.24951 • 1♀; Yuba Co., Marysville Buttes [Sutter Buttes]; 2.v.1928; H.H. Keifer leg.; CASENT8568092.

#### Non-type material examined, adults.

Canada – **Ontario** • 1♀; Ottawa, 40 km W, Almonte; 7.ix.1992; Kauri Mikkola leg.; alvar, ad lucem; Genitalia slide EvN4138; RMNH.INS.24138; MZH.

#### Non-type material examined, larvae.

United States – **California** • 9 larvae in ethanol; Marin Co., 400 Deer Valley Rd., San Rafael, Smith Ranch Homes; 15.iii.2017; S.J. Seybold, S. Swain leg.; ex collected stems and leaves, Marina strawberry tree, Arbutus × ‘Marina’ (*Arbutusunedo* × *A.andrachne*); RMNH.INS.31006, 31007, 31012–15 • 20 larvae (3–4^th^ instar); same collection data; 11.iv.2017; S.J. Seybold leg.; ex collected stems and leaves Marina strawberry tree; CSCA • 2 larvae; same collection data; from native *Arbutusmenziesii*; RMNH.INS.31008, 31009 • 2 larvae on slide (barcoded); Sonoma Co., 2900 Wild Turkey Run, near intersection of Bennett Valley Road and Savannah Trail; 855 ft [260 m]; 15.iii.2017; S.J. Seybold, S. Swain leg.; ex collected stems and leaves, manzanita, *Arctostaphylos* spp. (broad leaf); slides RMNH.INS.31005.P, RMNH.INS.31010.P • 1 larva in ethanol; same collection data; RMNH.INS.31011 • 28 larvae in ethanol; same collection data; Marina strawberry tree, Arbutus × ‘Marina’, (*Arbutusunedo* × *A.andrachne*); RMNH • 6 larvae in ethanol; same collection data; from manzanita, *Arctostaphylos* spp.; RMNH • 4 larvae (1–2^nd^ instar); 257 Perkins Street, Sonoma; 11.iv.2017; S.J. Seybold leg.; collected leaves Marina strawberry tree; CSCA.

#### Non-type material examined, leafmines.

possibly of *E.thoraceleuca*. United States – **Washington** • vacated mines; Washington, Chelan Co., Wenatchee National Forest, Entiat summit Road; 12.vii.2010; E.J. van Nieukerken leg.; EvN2010017; low *Pinusponderosa* forest on ridge, leafmines on *Arctostaphylosuva-ursi*; RMNH.INS.42859.

#### Additional online records – adults.

Canada – **Ontario** • 1♂, 1♀; Lambton Co., Port Franks; 21.viii.2021, 25.ix.2020; K. H. Stead leg.; BIOUG62319-H05, BIOUG74608-E04 (BOLD).

United States – **Arizona** • 1 adult; Cochise Co., Sierra Vista Southeast. Miller Canyon Upper Parking; 17.v.2022; Jim Eckert; https://www.inaturalist.org/observations/142023215. – **California** • 1 adult; San Diego Co., San Marcos; 31.vii.2018; Greg Smith leg.; https://bugguide.net/node/view/1568125 • 1 adult; Santa Clara Co., Silicon Valley, Stanford Academic Reserve; 5.vi.2021; Jen and Hilary Bayer leg.; Malaise trap; BIOUG92695-A02 (BOLD) • 1 adult; Tulare Co., Ash Mountain; 06.vii.2018; Graham Montgomery leg.; https://bugguide.net/node/view/1569217.

#### Additional online records – leafmines.

United States – **California** • Colusa Co., various localities; mines on *Arctostaphylosmanzanita*; 27.ii.2020, 4–5.ii.2021, 27, 28.iii.2021; K. Schneider leg.; https://www.inaturalist.org/observations/72384123, https://www.inaturalist.org/observations/72335843, https://www.inaturalist.org/observations/72447513, https://www.inaturalist.org/observations/39306497, https://www.inaturalist.org/observations/69086879, https://www.inaturalist.org/observations/69119629 • Contra Costa Co., Mount Diablo State Park; leafmines on Arctostaphylosmanzanitasubsp.laevigata; 8.ii.2020; Ken-Ichi Ueda leg.; https://www.inaturalist.org/observations/38489869 • Los Angeles Co., Claremont, California Botanic Garden; 2 leafmines on *Arctostaphylosinsularis*; Steven Kurniawidjaja leg.; https://www.inaturalist.org/observations/150883693 • Los Angeles Co., La Cañada Flintridge, Descanco Gardens; 2 leafmines on *Arbutus*; Steven Kurniawidjaja leg.; https://www.inaturalist.org/observations/155422286 • Marin Co., Santa Venetia, leafmine on unidentified host; 28.i.2020; Krissa Klein leg.; https://www.inaturalist.org/observations/38138633 • Modoc Co., FR-73, 0.1 road mi W Householder Reservoir entrance road; leafmines on *Arctostaphylosmanzanita*; 17.v.2021; K. Schneider leg.; https://www.inaturalist.org/observations/79232102, https://www.inaturalist.org/observations/79232103 • San Diego Co., 31.iii.2019; leafmines on *Arctostaphylosrainbowensis*; James Bailey leg.; https://www.inaturalist.org/observations/21914712 • San Diego Co., Cleveland National Forest; 11.xi.2021; leafmines on *Arctostaphylos*; Jorge Ayón leg.; https://www.inaturalist.org/observations/100888480.

#### Diagnosis.

*Etainiathoraceleuca* is easily recognized by the combination of a white thorax and the silver markings: a fascia and costal plus dorsal spot. Some *Stigmella* species have a similar pattern, but can be recognized by the distinct collar, comprising lamellar scales. Most similar are some species of *Acalyptris*, including the eastern *A.thoracealbella* (Chambers, 1873). This species has the pattern not so silvery, the antennae are paler, and the wings are narrower; moreover, the distribution does not seem to overlap much, but genitalia should be checked when in doubt. The male genitalia are characteristic of the genus *Etainia* by the valval apodemes, absent uncus, and structure of the phallus; it differs from *E.ochrefasciella* and *E.sericopeza* by the different shapes of the valva and gnathos, and the latter are very wide in *E.sericopeza* and very narrow in *E.ochrefasciella*. The female genitalia differ especially by the different structure of tergite 8.

#### Description.

**Figures 1–4.**
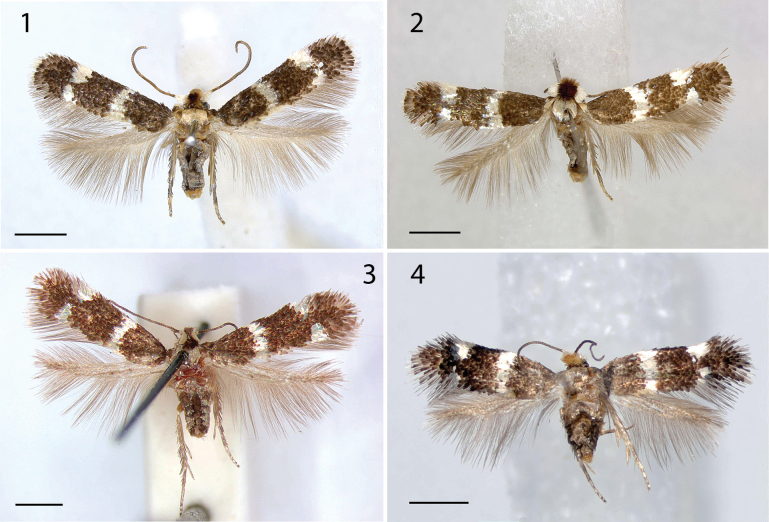
*Etainiathoraceleuca*, adult habitus **1** male Holotype AZ, genitalia slide EvN4950 **2** male, CA, Siskiyou Co., genitalia slide EvN4951, RMNH.INS.24951 **3** female, CA, Santa Barbara Co., San Marcos Pass, 7.vii./1965 **4** female, Canada, ON, Ottawa, genitalia slide EvN4138. Scale bars: 1 mm.

**Male** (Figs 1, 2). Forewing length 2.6–3.2 mm (2.9 ± 0.2, 7), wingspan 5.8–7.0 mm. Head: frontal tuft ferruginous, mixed with fuscous on frons, almost black on vertex, ferruginous in Canadian specimens, collar inconspicuous, comprising ferruginous or fuscous hair-scales. Scape and pedicel cream white. Flagellum fuscous, antenna with 49–57 segments (51.3 ± 2.9, 6), ratio to forewing length 16–19 (17.8 ± 1.4, 6) segments/mm. Thorax creamy white, tegulae either white or concolorous with forewing, forewing shining fuscous, pattern silvery white, consisting of a fascia at 1/3, and opposite costal and dorsal spots at 2/3, terminal fringe white beyond distinct fringe line, underside dark fuscous, no androconial scales present. Hindwing grey, a row of costal bristles behind frenulum, underside grey. Abdomen grey brown, genitalia with ochreous vestiture.

**Female** (Figs 3, 4). Forewing length 2.6–3.4 mm (3.0 ± 0.3, 6), wingspan 5.9–7.8 mm. Antenna with 41–49 segments (43.5 ± 3.7, 6), ratio to forewing length 8–11 segments/mm. As male, but frontal tuft completely ferruginous in Canadian specimens. Hindwing with costal bristles.

**Figures 5–8.**
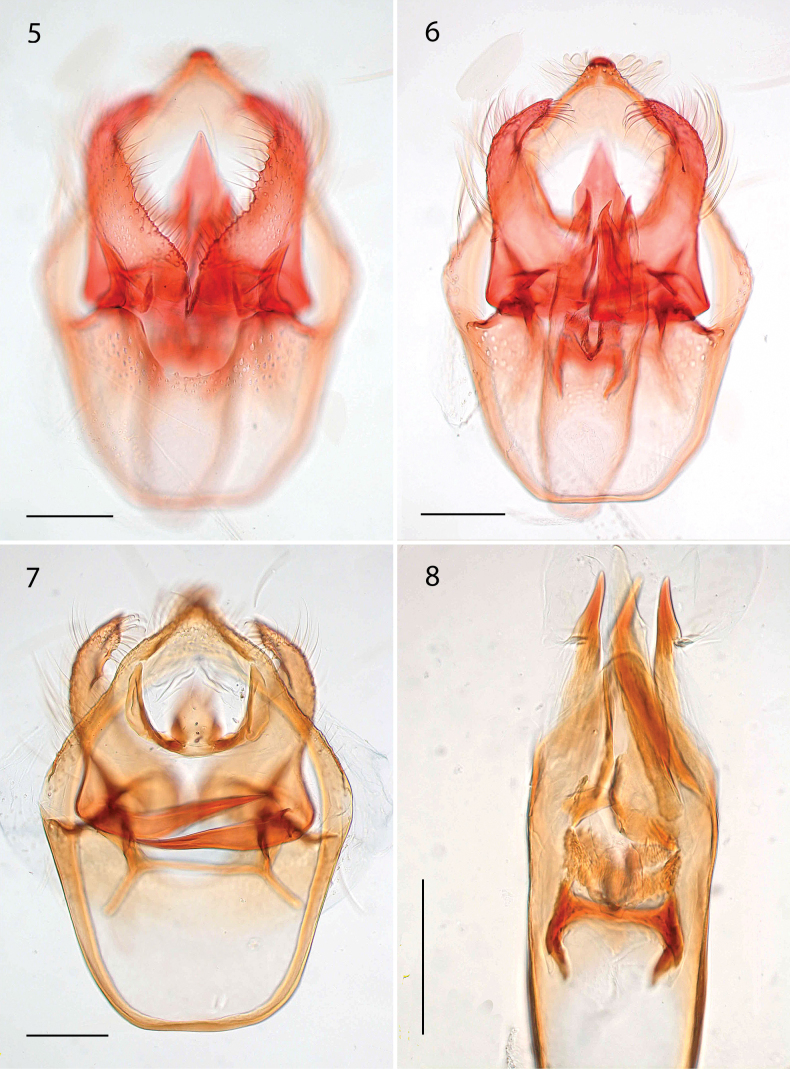
*Etainiathoraceleuca*, male genitalia **5, 6** holotype, AZ, genitalia slide EvN4950, respectively more ventrally and more dorsally focused **7, 8** CA, genitalia slide EvN4951, capsule, focused at valval processes and separate detail of phallus. Scale bars: 100 µm.

**Figures 9–12.**
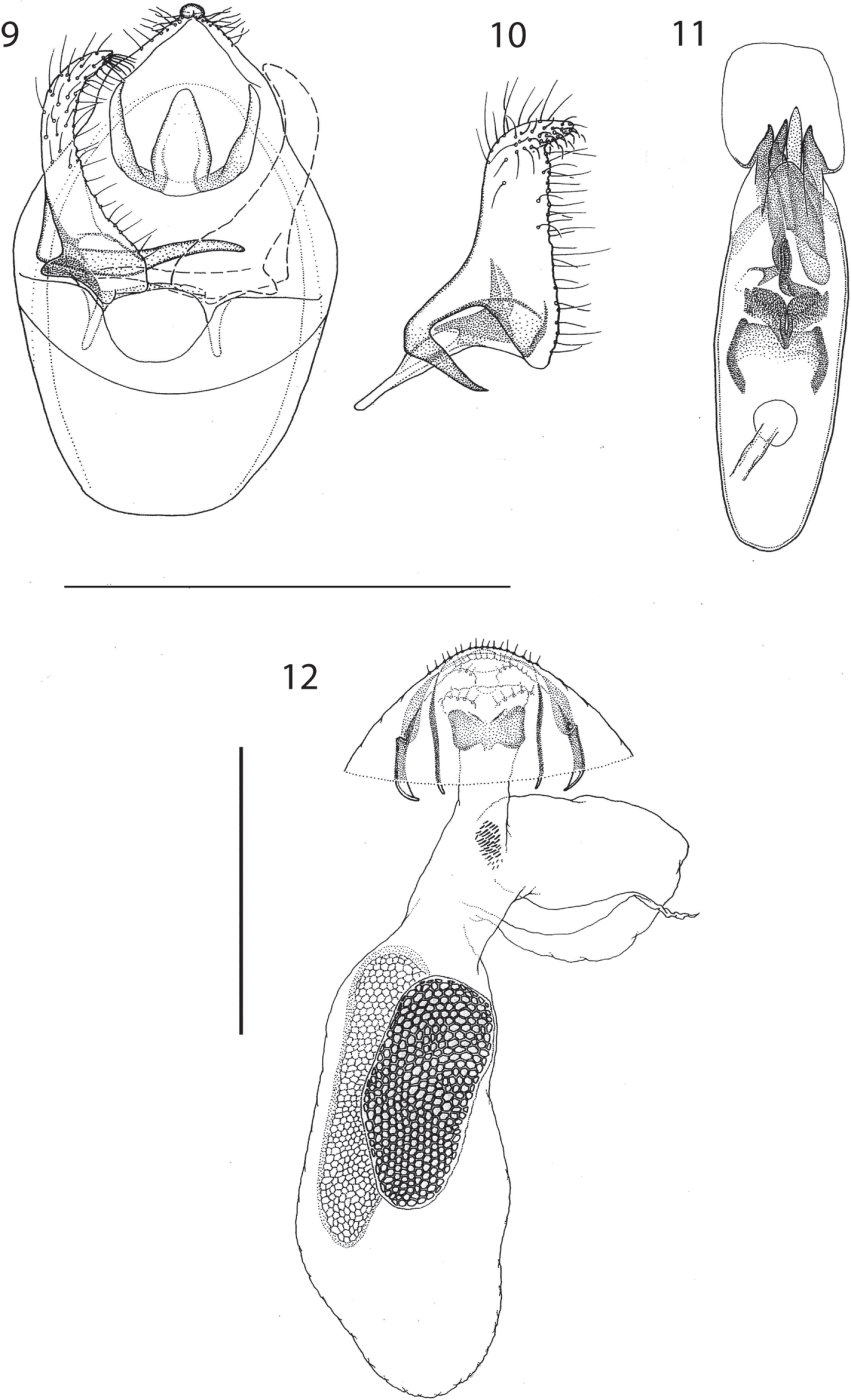
*Etainiathoraceleuca*, genitalia **9, 10** male genitalia slide USNM20877, phallus omitted, right valva illustrated separately, internal view **11** phallus, genitalia slide USNM20961 **12** female genitalia, genitalia slide USNM17493. Scale bars: 0.5 mm.

***Male genitalia*** (Figs 5–11). Capsule length 435–660 μm (544.0 ± 79.8, 6), 1.3–1.6× as long as wide. Vinculum large, anteriorly truncate, posteriorly with U-shaped excavation. Tegumen produced into triangular, pointed pseuduncus, uncus absent. Gnathos with median lobe relatively broad at base, pointed at tip, length less than twice its width. Valva length 200–320 μm (255.9 ± 38.2, 6), relatively narrow, curved and tapering slightly to an abruptly down-curved apex; dorsal apodeme greatly elongate, sharply curved, and smooth; transtilla transverse bar straight, rather long, sublateral processes distinct, about half length of transverse bar. Phallus 325–460 μm (405.5 ± 49.0, 6), 2.9–3.4× as long as wide; apically ending in a long tongue-shaped process dorsally, and a pair of ventral pointed carinae; vesica with the following sclerotizations: basally an H-shaped sclerotization, followed by a distinct striate cathrema and an indistinct complex sclerotized structure, ending in a single large pointed cornutus protruding from the phallus.

**Figures 13–17.**
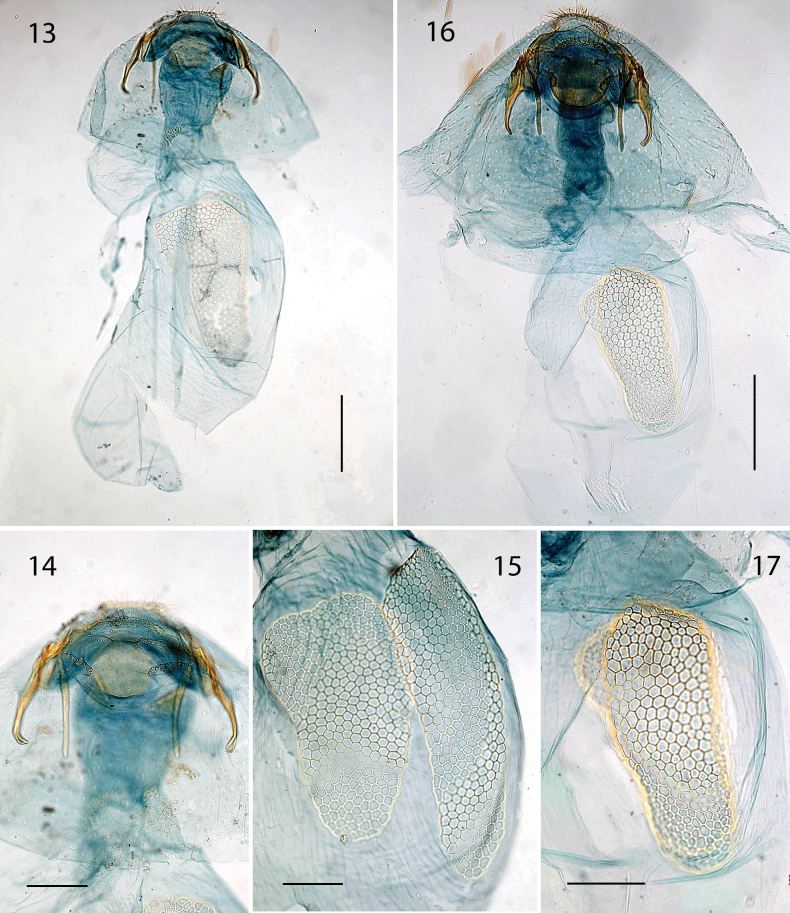
*Etainiathoraceleuca*, female genitalia **13, 14** CA, genitalia slide DRD3401, with detail of terminal segments, dorsally **15** CA, genitalia slide DRD3329, detail signa **16, 17** ON, genitalia slide EvN4138, with detail of signa. Scale bars: 200 µm (**13, 16**); 100 µm (**14, 15, 17**).

***Female genitalia*** (Figs 12–17). Abdominal end broadly rounded, anal papillae a narrow band with more than 50 setae in total; T8 with pointed posterior margin; on T8 three transverse broken rows with groups of socketed setae, posteriorly two connected groups of ca 21–25 setae each; medially two widely separate groups of 12–13 setae at either side; anteriorly two groups of ca 14–17 setae. Anterior apophyses widely separate, with curved tips; posterior apophyses straight. T7 with medial indentation in posterior margin. Corpus bursae total length ca 710–855 μm. Ductus bursae with a paired sclerotized structure near cloaca and group of small spines laterally, more anteriorly; corpus bursae with paired elongate reticulate signa, usually different in length, longest 520–550 μm; ca 10–12 cells wide, shortest 360–425 μm, ca 10–17 cells wide. Ductus spermathecae with 2 indistinct convolutions.

**Egg.** In the few examples seen on leaf underside, the usual domed egg scale of Nepticulidae. When the mine develops, the egg is more or less in the center of the leaf spot.

**Larva** (Figs 18–40, 45–52). First instar: head-capsule width 0.24 mm, length 0.27 mm, (overall length 2.78 mm) (*n* = 1), Second instar: head-capsule width 0.33–0.36 mm, length 0.35–0.37 mm, (overall length 4.21–4.59 mm) (*n* = 3); Third instar: Head-capsule width 0.47–0.48 mm, length 0.42–0.47 mm, (overall length 1.71–2.06 mm) (*n* = 6); Final (fourth) instar: head-capsule width 0.44–0.56 mm, length 0.41–0.53 mm, (overall length 2.63–4.71 mm) (*n* = 11).

**Figures 18–21.**
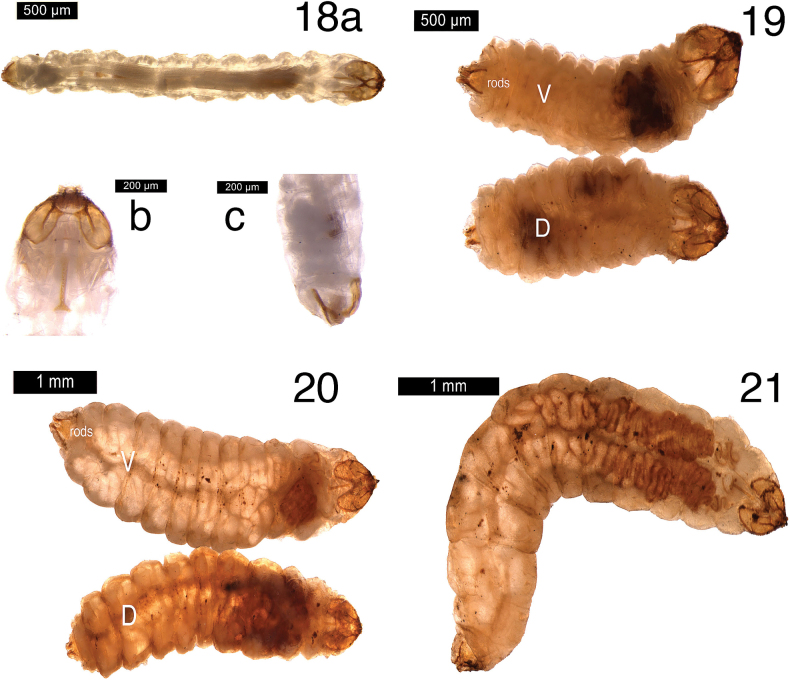
Larval whole body of *Etainiathoraceleuca***18** first instar: **a** whole larva posterior (L) to anterior (R), dorsal view **b** close up head (ventral view) **c** close up posterior (dorsal view) **19** second instar (dorsal and ventral) **20** third instar (dorsal and ventral) **21** fourth instar (ventral-lateral).

**Figures 22–28.**
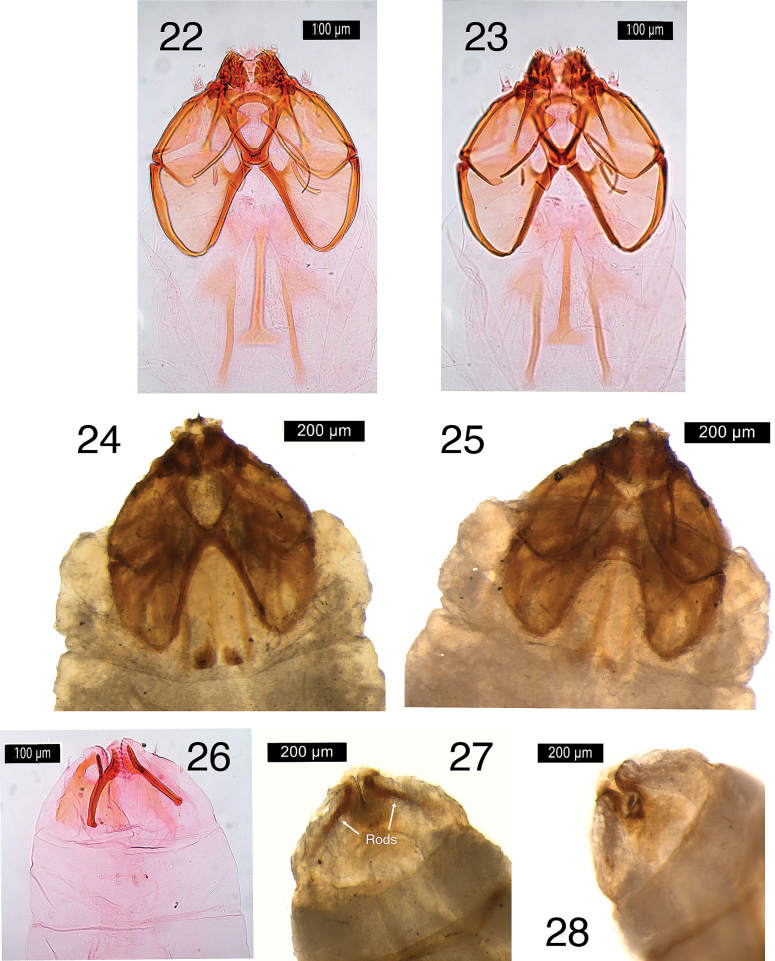
Larval head and posterior end of *Etainiathoraceleuca***22, 23** head capsule of instar 3? in slide, dorsal (**22**) and ventral (**23**) view, RMNH.INS.31005.P (DNA barcoded larva) **24, 25** head of instar, untreated larva, dorsal (**24**) and ventral (**25**) view **26–28** posterior segments **26** in slide RMNH.INS.31005.P **27** ventral **28** dorsal.

**Figures 29–40.**
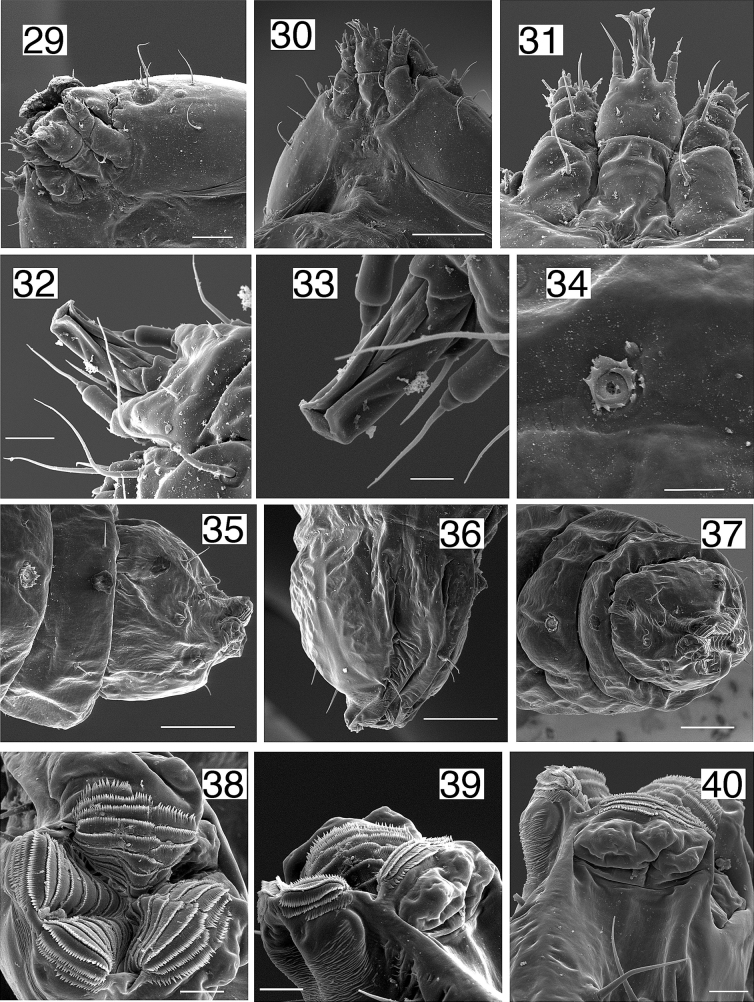
*Etainiathoraceleuca*, larva, scanning electron microscopy [scale bars = µm] **29** early instar head ventro-lateral **30** early instar head ventral **31** early instar labium with spinneret, labial palps and maxillae with palps, ventral **32** mid instar dorsal spinneret and labial palps **33** mid instar dorsal spinneret **34** early instar spiracle A4 **35** mid instar A8-A10 lateral **36** mid instar lateral A10 **37** mid instar A8–A10 posterior lateral **38** early instar rows of spines in anal opening A10 **39** early instar ventral A10 **40** early instar lateral ventral A10. Scale bars: 100 µm (**30, 35–37**); 50 µm (**29, 31**); 20 µm (**32, 34, 38–40**); 10 µm (**33**).

**Figures 41–44.**
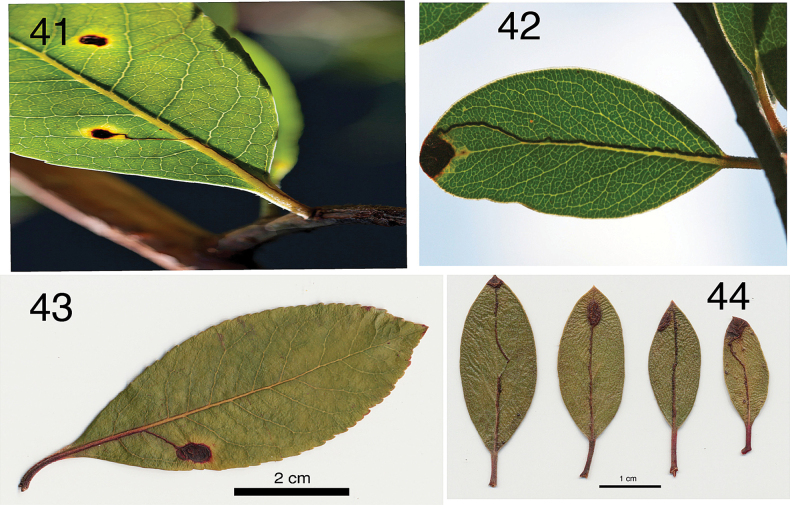
Leafmine damage caused by *Etainiathoraceleuca***41** Marin Co., CA: S. Swain, leafmine on Marina strawberry tree, *Arbutus* × ‘Marina’ **42** CA: Sonoma Co., S. Swain, leafmine on manzanita, *Arctostaphylos* spec. **43** CA: San Diego Co., S.J. Seybold, MA Siefker, coll., leafmine on Marina strawberry tree, *Arbutus* × ‘Marina’ **44** CA: Marin Co., S.J. Seybold, coll., mines on stems and leaves from manzanita, *Arctostaphylos* spec.

**Figures 45–53.**
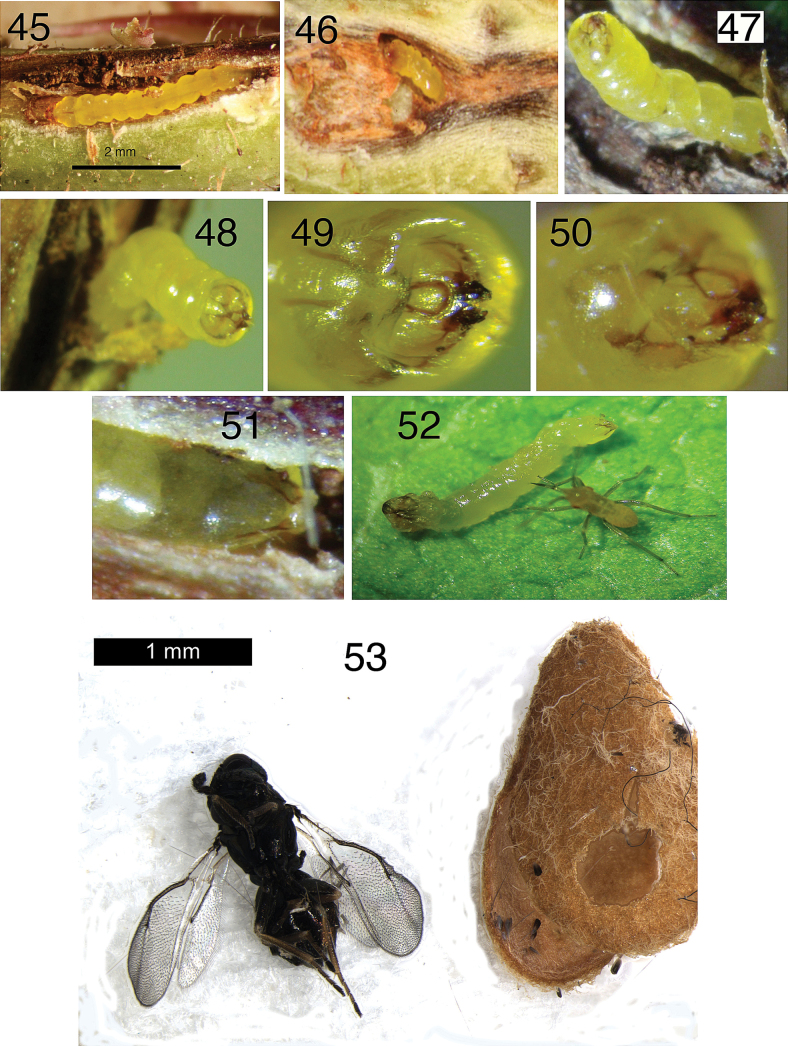
Active larvae of *Etainiathoraceleuca* boring in woody stems of Marina strawberry tree, *Arbutus* × ‘Marina’ from CA: Marin Co. unless otherwise mentioned **45–46** CA: Sonoma Co., S. Swain **47–50** open stem with larval head, dorsal view **51** larva in stem with anal rods visible **52** larva, removed from stem, attacked by a mirid nymph **53** Chalcidoid parasitoid, most likely Eulophidae: Entedoninae and cocoon of *Etainiathoraceleuca* with exit hole of parasitoid.

**Pupa** (skin) (Figs 56, 57). 2.2 mm long, 0.9 mm wide.

**Figures 54–57.**
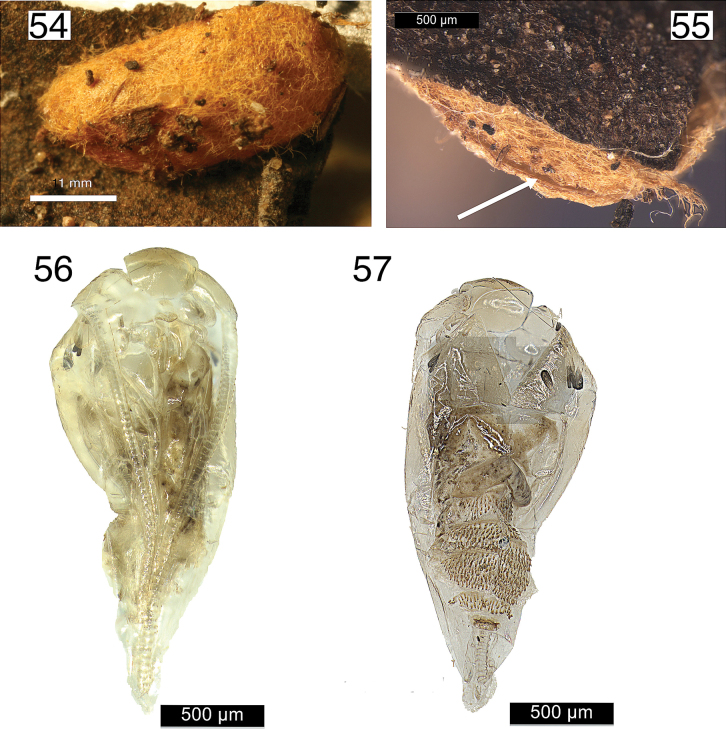
Cocoons (54–55) and pupal skin (56–57) of *Etainiathoraceleuca*.

**Cocoon** (Figs 53–55). Yellow-brown and ovoid, 2.5–3.1 mm long and 1.5 mm wide. Emergence slit about half the length along the narrow periphery.

#### Biology.

**Hostplants.**Ericaceae: *Arbutusarizonica* (A. Gray) Sarg. (Arizona madrone), *Arbutusmenziesii* Pursh (Pacific madrone), *Arbutus×andrachnoides* Link ‘Marina’, (Marina strawberry tree, possibly *Arbutusunedo* L. × *A.andrachne* L.), *Arctostaphyloscanescens* Eastw. (Eiseman 2022), *Arctostaphylos ?glauca* Lindl., *Arctostaphylosinsularis* Greene & Parry (Island manzanita), *Arctostaphylosmanzanita* Parry (Manzanita), Arctostaphylosmanzanitasubsp.laevigata (Eastw.) Munz, *Arctostaphylospungens* Kunth (Eiseman 2022), *Arctostaphylosrainbowensis* J. E. Keeley & Massihi, most likely also *Arctostaphylosuva-ursi* (L.) Spreng. (Bearberry).

**Life cycle.** Eggs are apparently laid singly on the underside of leaves in late summer to early fall, where they remain quiescent for several weeks. In late fall, the larvae hatch and begin burrowing into leaf tissue. On the oviposition site there develops a red to black stained leaf spot; from there the larvae make a thin, linear mine running along the lateral and midveins of the hostplants’ leaves, often very straight or sometimes with a few loops; the frass line is central and almost completely fills the gallery; the mined area soon turns red to black. Larvae continue burrowing through the petiole and into subtending twigs where they mine phloem tissues. After feeding in the twig cambium for several months, larvae bore their way out of the twigs and drop into the leaf litter beneath the plant. The exit holes are little slits in the twig epidermis, resembling those of other nepticulids. On California’s north coast, this occurs from April to May. Larvae pupate in the duff, tying dead leaves and perhaps other organic material together with bright-saffron to orange silken cocoons. Pupation is complete by June with most adult moths emerging early to mid-May in 2019 and 2020. Moths were found between 26 April and 14 October, which may indicate two annual generations, or a rather irregular emergence of adults. Adults are usually found at light.

#### Damage details

**(Figs 41–44).** Initial damage symptoms appear as inconspicuous black spots on the hostplant leaf blade, caused by a larva tunneling in a wandering circular pattern (Fig. 41). The circles gradually enlarge until the larva encounters a lateral leaf vein. The larva then normally follows the lateral vein down to the midvein, through the petiole (Figs 41–44). Damage by the larvae can be seen on the undersides (and sometimes the top) of the leaf as a thin but conspicuous red to black line marking the tunnel. Sometimes a larva will follow the lateral vein to the edge of the leaf, and then circle toward the leaf center until it again finds a lateral vein. Once the larva reaches the thicker tissues of the petiole and begins boring into the twig’s cambial tissues the line gradually disappears. Twig bark excision with a scalpel will reveal substantial feeding damage in the cambium. Undamaged leaves of *Arbutus* normally last at least one season, sometimes a few months more (Berner and Law 2016: table 2). All hostplants observed so far dropped their damaged leaves before the end of their first season.

As larvae from many leaves migrate into subtending twigs to feed, their numbers and associated damage become concentrated, damaging the cambium to such an extent that the distal portions of the twig are frequently killed. These branches wilt and die rapidly, leaving the wilted end of the twig to droop, shrivel, and then harden into a shepherd’s crook. Even when twigs are not killed, the bark splits and callus tissue will form on the twigs, disfiguring and sometimes distorting it. In severe cases, young *Arbutus* ‘Marina’ trees have been killed, and older trees rather severely disfigured, with less than 50% live canopy remaining. Damage to native manzanita and madrone does not appear to be as severe.

#### Parasitoids.

As might be expected for a moth in its native range, parasitoid wasp pupae have been found inside the mines on occasion. One Chalcidoidea parasitoid (most likely Eulophidae, Entedoninae, C. Eiseman pers. comm.) emerged in summer 2023 from cocoons from Sonoma (near Napa Co.) found by SVS on 11 May 2023; it made a circular exit hole on a broad cocoon surface (Fig. 53). A moth emerged from another cocoon from the same date and location during the same summer.

#### Distribution

**Figures 58–59.**
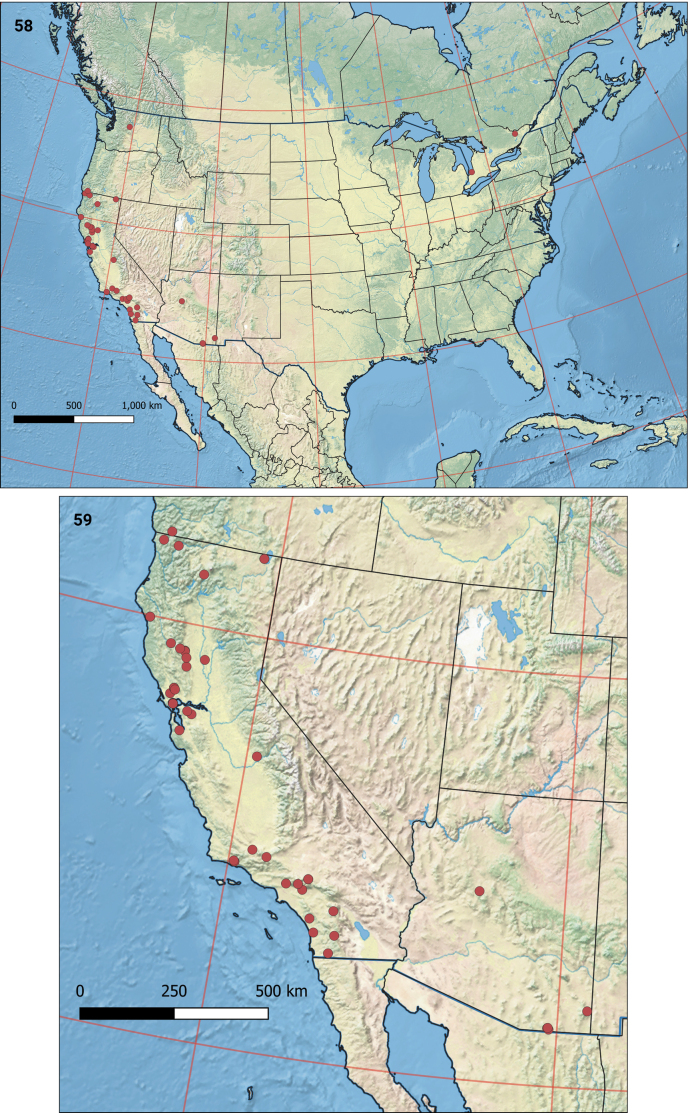
Distribution of *Etainiathoraceleuca***58** in North America **59** in Oregon, California and Arizona.

**(Figs 58, 59).** Most records are from California, where *E.thoraceleuca* has been found over much of the coastal ranges of California and collected from Del Norte County in the extreme northwestern part of the state to as far south as San Diego County, in adjacent Oregon (leafmines, Josephine Co., Rough and Ready Botanical Wayside, 42.0959, −123.6831; C. Eiseman pers. comm.), in Arizona in the Huachuca mountains (type locality), Chiricahua mountains (leafmines on *A.pungens*, Cochise Co., Cave Creek Canyon, Cathedral Vista Trail, 31.8866, −109.1721, C. Eiseman pers. comm.; illustrated by Eiseman 2022), Yavapai County, and three specimens from Ontario, Canada. Leafmines collected in Washington State also probably belong to this species.

#### DNA barcodes

**Figure 60.**
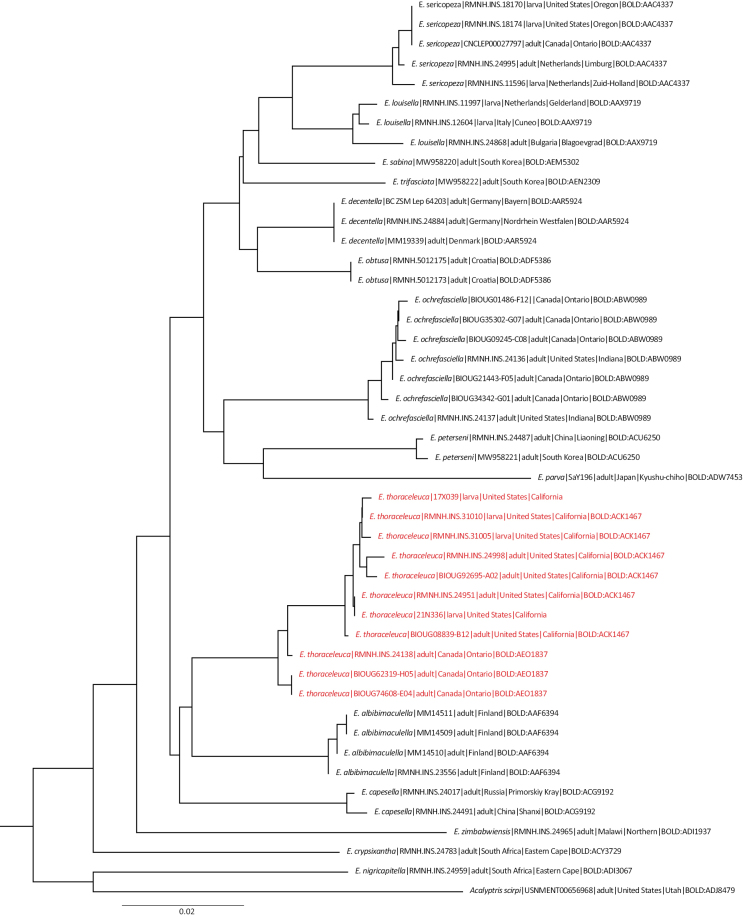
Neighbor-joining tree of DNA barcodes (mt gene COI-5P) of *Etainia* species, using the Kimura distance model. *Acalyptrisscirpi* (Braun, 1925) is used as outgroup. Labels show species name, Sample ID, life stage, Country, State or Province and BIN (Barcode Identification Number).

**(Fig. 60).** We have DNA barcodes from eleven specimens, four larvae and seven adults: seven full barcodes belonging to BINBOLD:ACK1467 with an average KP2 distance of 0.39%, and a maximum distance of 0.83%, and three barcodes of the Canadian specimens belonging to BINBOLD:AEO1837 (no variation) at a distance of 1.5% to the nearest Californian sequence (Fig. 58). Unfortunately, we failed to amplify COI from the holotype.

The identical barcodes of adults and larvae undoubtedly show that the described larvae and damage belong to the same species as the adults.

#### Remarks.

The specimens from Canada differ in having a completely orange head and in their female genitalia; in both specimens the signa are almost equal length, 360 μm. In a Californian female specimen one of the three rows of setae on T8 seems to be missing. The genitalia of the male Canadian specimen could not yet be examined. For now, we consider the Canadian specimens as belonging to the same species, but further study and material is required to evaluate the possibility of hidden diversity.

#### Etymology.

The specific name, *thoraceleuca*, a noun in apposition, is derived from the Greek noun *thorax* (breastplate) and adjective *leukos* (white), referring to the characteristic white thorax.

## ﻿Discussion

### ﻿Phylogeny

In a maximum-likelihood analysis of Nepticulidae based on a maximum of eight genes (to be published elsewhere), *E.thoraceleuca* groups with *E.albibimaculella* and the eastern Palearctic *E.capesella* (Puplesis, 1985) as sistergroup to all other Holarctic representatives of *Etainia*, which are probably all associated with *Acer* (Sapindaceae). The grouping of *E.albibimaculella* and *E.capesella* was also observed earlier (Doorenweerd et al. 2016), and since both *E.thoraceleuca* and *E.albibimaculella* feed on Ericaceae, it may indicate that *E.capesella* is also an Ericaceae feeder.

The sister-group relationship between *E.thoraceleuca* and *E.albibimaculella* may suggest a scenario where the common ancestor invaded North America from Asia, where it continued on the same hostplant, *Arctostaphylosuva-ursi* and from there invaded the mountains in the West and expanded its host range to include the abundant other Ericaceae hostplants already present.

### ﻿Distribution

The three Canadian records, far away from most records from the western USA, suggest that the species may have a continuous distribution with the northern *Arctostaphylosuva-ursi*, which has a very wide distribution throughout Canada and the northern and western United States. It is remarkable, however, that this relatively large and conspicuous nepticulid has not been recorded anywhere else in Canada, and also not in the extensive Canadian Malaise trapping programs (Hebert et al. 2016). In fact, *E.thoraceleuca* has only been collected twice in a Malaise trap, the specimens cited here as BIOUG08839-B12 from San Diego County and BIOUG92695-A02 from Santa Clara County. The apparent rarity is comparable to that of the European *E.albibimaculella*, for which records outside Finland and Sweden are extremely rare (van Nieukerken and Johansson 1990; Roweck and Savenkov 2002; Mazurkiewicz 2007; Kopp 2010; Nel et al. 2020), despite the likewise large distribution of bearberry in Europe and Asia.

### ﻿Hostplant relationships

The genera *Arbutus* and *Arctostaphylos* are closely related and grouped in the subfamily Arbutoideae (Kron et al. 2002), and many insect species share these genera as hosts. This is also the case for several leafminers: *Coptodiscaarbutiella*, *Marmaraarbutiella*, *Coleophoraglaucella* Walsingham, 1882, *Epinotianigralbana* (Walsingham, 1879), *Epinotiaterracoctana* (Walsingham, 1879), and an undescribed *Stigmella* species (Eiseman 2022). The oligophagy of *Etainiathoraceleuca* thus follows this common pattern. It should be searched for on *Arbutusxalapensis* in New Mexico and Texas and is likely to be found on many more *Arctostaphylos* species.

### ﻿Impact

As *Etainiathoraceleuca* is indigenous in California, its occurrence on cultivated plants was to be expected and by itself not of much concern. In some cases, however, the damage made by the insect can become a nuisance, but the use of the neonicotinoid imidacloprid in many nurseries (personal communications to SVS) has probably prevented this from becoming problematic. This, however, is in no way a plea to use these pesticides against this insect, as the damage caused by neonicotinoids to insects, especially bees, is too well known (Pisa et al. 2021). It is better to allow the native parasitoids and predators to do their work. Care should, however, be taken to avoid distributing infested plant material outside the native area, to avoid the possibility that *E.thoraceleuca* could become established elsewhere and might become invasive.

## Supplementary Material

XML Treatment for Etainia thoraceleuca

## ﻿Additional information

### Author ORCIDs

Erik J. van Nieukerken https://orcid.org/0000-0002-5721-1840

Marc E. Epstein https://orcid.org/0000-0001-8659-5815

### Data availability

The data that support the findings of this study are available in the main text and in datasets https://www.gbif.org/dataset/42e8fdc3-bcff-45ba-b318-605173ab1f04 and https://doi.org/10.5883/DS-ETAARB.
